# Electrically induced ambipolar spin vanishments in carbon nanotubes

**DOI:** 10.1038/srep11859

**Published:** 2015-07-07

**Authors:** D. Matsumoto, K. Yanagi, T. Takenobu, S. Okada, K. Marumoto

**Affiliations:** 1 Division of Materials Science, University of Tsukuba, Tsukuba, Ibaraki 305-8573, Japan; 2 Department of Physics, Tokyo Metropolitan University, Hachioji, Tokyo 192-0397, Japan; 3 Department of Applied Physics, Waseda University, Tokyo 169-8555, Japan; 4 Division of Physics, University of Tsukuba, Tsukuba, Ibaraki 305-8573, Japan; 5 Japan Science and Technology Agency (JST), PRESTO, Kawaguchi, Saitama 322-0012, Japan; 6 Tsukuba Research Center for Interdisciplinary Materials Science (TIMS), University of Tsukuba, Tsukuba, Ibaraki 305-8571, Japan

## Abstract

Carbon nanotubes (CNTs) exhibit various excellent properties, such as ballistic transport. However, their electrically induced charge carriers and the relation between their spin states and the ballistic transport have not yet been microscopically investigated because of experimental difficulties. Here we show an electron spin resonance (ESR) study of semiconducting single-walled CNT thin films to investigate their spin states and electrically induced charge carriers using transistor structures under device operation. The field-induced ESR technique is suitable for microscopic investigation because it can directly observe spins in the CNTs. We observed a clear correlation between the ESR decrease and the current increase under high charge density conditions, which directly demonstrated electrically induced ambipolar spin vanishments in the CNTs. The result provides a first clear evidence of antimagnetic interactions between spins of electrically induced charge carriers and vacancies in the CNTs. The ambipolar spin vanishments would contribute the improvement of transport properties of CNTs because of greatly reduced carrier scatterings.

Carbon nanotubes (CNTs) have attracted considerable attention because they exhibit various excellent properties, such as ballistic transport; high charge mobility due to the ballistic transport was observed in field-effect transistors (FETs)^1^. Depending on their chirality, single-walled CNTs (SWCNTs) are either metallic (zero band gap) or semiconducting with band gaps^2^,^3^. The structure-property correlation between SWCNTs and the resultant unique diversity in their electronic properties has allowed the fabrication of devices that are superior in performance and functionality to silicon-based devices^1^,^4^,^5^,^6^,^7^,^8^,^9^. To understand the nature of the ballistic transport completely, a study of spin states in CNTs is indispensable because such spins would cause carrier scattering due to magnetic interactions and prevent the realization of the ballistic transport. In particular, a microscopic investigation of spin states under device operation is crucially important because it would clarify the electronic states of electrically induced charge carriers from a microscopic viewpoint which directly relate to the device performance. Calculations with spin-polarized density functional theory (DFT) have shown the spin formation at atomic vacancies in SWCNTs^10^. The atomic vacancies with spins are considered to be intrinsically formed in CNTs^10^. However, the relation between the spin formation and the device performance have not yet been investigated. Such investigation would provide an insight for the mechanism of the realization of the ballistic transport.

A most effective method to study the spin states in CNTs under device operation is to utilize an electron spin resonance (ESR) spectroscopy using transistor structures^11^,^12^,^13^,^14^,^15^,^16^. This technique is an extremely clean method because it can directly observe electrically induced charge carriers without modifying CNTs chemically; the ESR method has clarified various microscopic properties, such as spin states and local structures of materials and devices^11^,^12^,^13^,^14^,^15^,^16^. Although the ESR method have been expected to clarify the spin states in CNTs under device operation for a decade, one could not perform the ESR study of CNT devices because of experimental difficulties, such as low charge-accumulation ability using usual solid insulators and dielectric loss due to metallic CNTs with thick film thickness.

In this work, we present an ESR study of CNT transistors under device operation. We adopted an ion-gel dielectric in transistor structures to overcome the low charge-accumulation ability^13^,^14^,^17^,^18^,^19^,^20^,^21^,^22^,^23^,^24^. High-quality thin films of semiconducting SWCNTs were utilized to avoid the dielectric loss due to metallic CNTs^25^,^26^,^27^. From simultaneous measurements of the ESR and the transistor characteristics, we observed a clear correlation between the ESR decrease and the current increase under high charge density conditions in the CNT materials for the first time. We directly discovered electrically induced ambipolar spin vanishments in the SWCNTs. Our result provides a first clear evidence of antiferromagnetic interactions between spins of electrically induced charge carriers and vacancies in the SWCNTs. The ambipolar spin vanishments would contribute the improvement of transport properties of CNTs because of greatly reduced carrier scatterings due to magnetic interactions.

## Results and Discussion

### Dependence of ESR of SWCNT transistors on gate voltages

**Figure 1:**
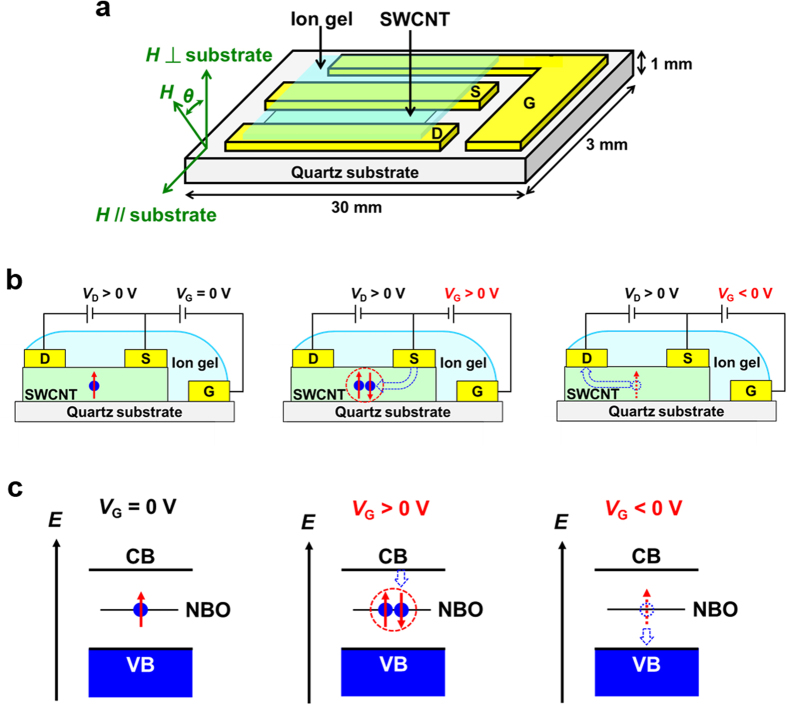
Schematics of a SWCNT transistor and spin states in SWCNTs. (**a**) Schematic of the device structure of the SWCNT transistor used in this study. (**b**) Schematics of the cross section of the device structure with spin states in the transistor at *V*_G_ = 0 V (left), for *V*_G_ > 0 V (center), and for *V*_G_ < 0 V (right). (**c**) Schematics of the energy diagram of conduction band (CB), valence band (VB), and non-bonding orbital (NBO) of the SWCNTs with spin states at *V*_G_ = 0 V (left), for *V*_G_ > 0 V (center), and for *V*_G_ < 0 V (right).

**Figure 2:**
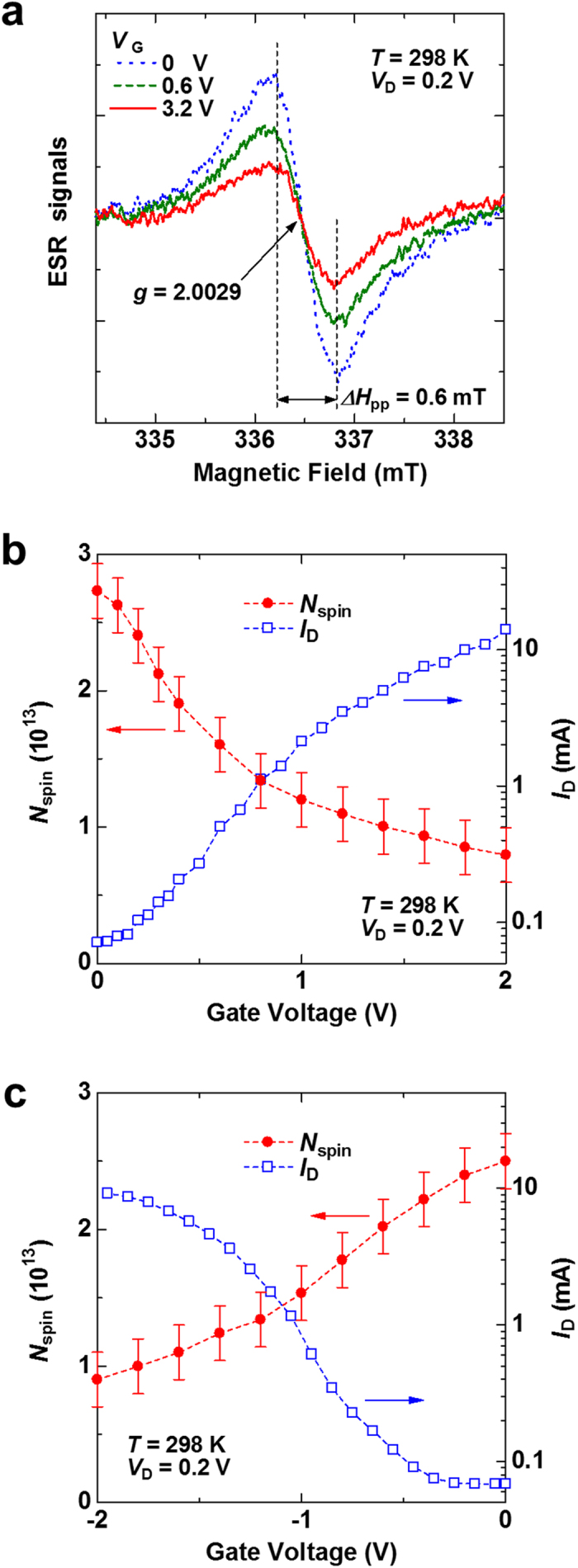
Electrically induced ambipolar spin vanishments in the SWCNT transistor. (**a**) ESR signals of the SWCNT transistor at *V*_G_ of 0 V (blue dotted line), 0.6 V (green dashed line), and 3.2 V (red solid line), where *V*_D_ = 0.2 V at the external magnetic field *H* perpendicular to the substrate (*H*_⊥_) at 298 K. (**b**,**c**) Dependence of the number of spins, *N*_spin_, and of the *I*_D_ of the SWCNT transistor on *V*_G_ for (**b**) *V*_G_ ≥ 0 V and (**c**) *V*_G_ ≤ 0 V, where *V*_D_ = 0.2 V at 298 K at *H*_⊥_. The *I*_D_ data are plotted using a semilogarithm scale.

First, we present the ambipolar spin vanishments in the SWCNTs by applying gate voltage (*V*_G_). Figure 1a shows the device structure of the SWCNT transistor. Ion gels used in the structure are currently attracting considerable attention because they enable low voltage operation due to the formation of an electric double layer (EDL) at the semiconductor/insulator interfaces^13^,^14^,^17^,^18^,^19^,^20^,^21^,^22^,^23^,^24^. Details of the fabrication of the SWCNT thin films and their devices are described in the Methods. Figure 2a shows the ESR signals of the SWCNT transistor at *V*_G_ values of 0, 0.6, and 3.2 V with a drain voltage *V*_D_ = 0.2 V. The observed ESR signals show a Lorentzian ESR lineshape with the *g* value of 2.0029 ± 0.00002 and the peak-to-peak ESR linewidth Δ*H*_pp_ of 0.6 ± 0.1 mT. These features do not depend on *V*_G_. The origin of the ESR signal is ascribed to atomic vacancies in the SWCNTs, as discussed later^10^. Notably, the ESR intensity decreased when *V*_G_ increased from 0 to 3.2 V.

### Electrically induced ambipolar spin vanishments in SWCNTs

To investigate the correlation between the ESR intensity and the transistor characteristics, we present the results of these simultaneous measurements as a function of *V*_G_. We use the number of spins, *N*_spin_, to present the ESR intensity of the SWCNT transistor, which was evaluated by integrating the ESR signal twice and by comparing the *N*_spin_ value with that of the Mn^2+^ marker sample assuming the Curie law. Figure 2b,c show the *V*_G_ dependence of *N*_spin_ and drain current (*I*_D_) of the SWCNT transistor in positive and negative *V*_G_ regions, respectively, which were simultaneously measured using the same device. The *I*_D_ increased as the absolute value of *V*_G_ increased in both positive and negative *V*_G_ regions, demonstrating ambipolar transistor operation, which are consistent with the results of previous studies^17^,^18^,^22^,^24^. Notably, in contrast to the *I*_D_ increase, *N*_spin_ clearly decreased as the absolute value of *V*_G_ increased in both positive and negative *V*_G_ regions. That is, we observed a clear correlation between the *N*_spin_ decrease and the *I*_D_ increase for both electron and hole accumulation in the SWCNT transistor. Therefore, we directly demonstrated electrically induced ambipolar spin vanishments in the SWCNTs. Such spin vanishments by *V*_G_ has not yet been reported. This result means the non-magnetization of vacancies’ spins, which is an unexpected result because *N*_spin_ of organic transistors always increases when |*V*_G_| increases owing to charge accumulation in devices^11^,^12^,^13^,^14^.

Next, we discuss the mechanisms of the electrically induced ambipolar spin vanishments. For positive *V*_G_ region, the spin vanishment is explained by the formation of the spin pairings between spins of vacancies and electrically induced electrons which are doped by *V*_G_ in the SWCNTs (Fig. 1b,c, center). Our result provides a first clear evidence of antiferromagnetic interactions between these spins. For negative *V*_G_ region, the spin vanishment can be explained by the discharge of vacancies’ spins due to hole doping by *V*_G_ (Fig. 1b,c, right-hand side). It should be noted that no change in the ESR signal was observed when changing *V*_D_. If the origin of the ESR signal is electrically induced charges, the ESR signal should change by changing *V*_D_ because the amount of electrically induced charges in a transistor channel depends on *V*_D_ under gradual-channel approximation^28^,^29^. Thus, no change in the ESR signal when changing *V*_D_ confirms that the observed ESR signal is not ascribed to electrically induced charges.

### No ESR observation in Tomonaga-Luttinger liquid

The reason for the non-observation of an ESR signal due to electrically induced charges is explained by the formation of Tomonaga-Luttinger-liquid (TLL) states^30^,^31^. On the basis of the framework of TLL theory with spin-symmetry breaking and electron-electron interaction in one-dimensional (1D) electron system, these previous studies have showed that the ESR linewidth due to TLL states becomes extremely broad, on the order of 100 mT, even at low temperatures such as 4 K, and further broadening of the ESR linewidth at higher temperatures^30^,^31^. Although we performed ESR measurements at low temperatures below 4.2 K with applied *V*_G_, we could not observe any additional ESR signal due to electrically induced charges because of the extremely broadening of the ESR linewidth mentioned above. Therefore, the present ESR study demonstrates the formation of TLL states in the SWCNT transistor from a microscopic viewpoint, even in the bundled SWCNTs with relaxed 1D character. We comment that the origin of the ESR signals reported in previous CNT studies should be ascribed to localized states (atomic vacancies, tube ends, etc.) and/or extrinsic impurities (catalysts etc.) rather than delocalized charge carriers in CNTs^30^,^32^,^33^,^34^,^35^,^36^,^37^,^38^,^39^,^40^,^41^,^42^,^43^,^44^,^45^.

### Microscopic properties of atomic vacancies in SWCNTs

**Figure 3:**
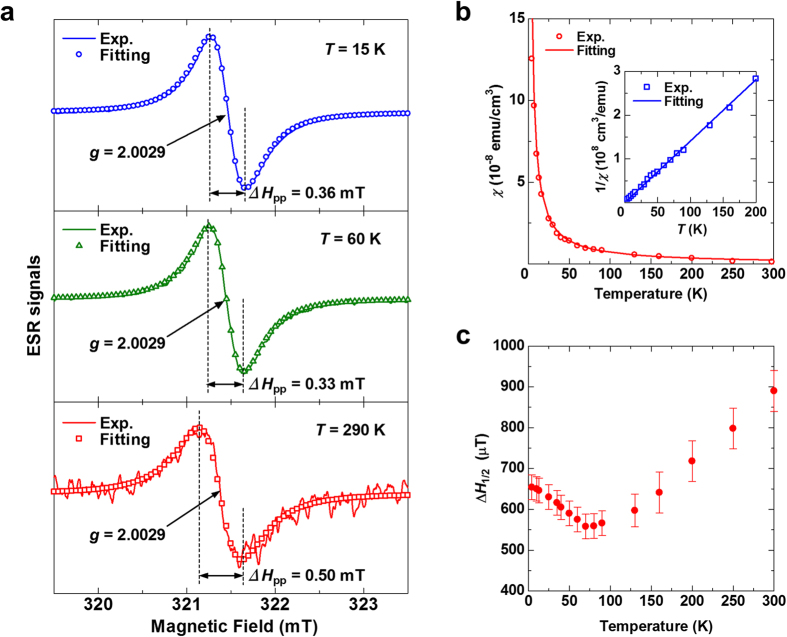
ESR study of a SWCNT thin film. (**a**) ESR signals of the SWCNT thin film at *H*_⊥_. The solid lines show the data measured at 15 K (upper), 60 K (center), and 290 K (bottom). The symbols of circles (upper), triangles (center), and squares (bottom) show the least-squares fits with a Lorentzian lineshape to the observed ESR signals. (**b**) Temperature dependence of the spin susceptibility *χ* (main panel) and the inverse spin susceptibility 1/*χ* (inset) of the ESR signal of the SWCNT thin film at *H*_⊥_. The solid lines show the least-squares fits with the Curie law to the data. (**c**) Temperature dependence of the full width at half maximum, Δ*H*_1/2_, of the ESR signal of the SWCNT thin film at *H*_⊥_.

In the following, we present further detailed microscopic properties of atomic vacancies, such as spin concentration, spin-lattice relaxation time, motion of vacancies, and anisotropy of spin-orbit interaction. We present the ESR results of SWCNT thin films with 300 nm thickness because the signal-to-noise (SN) ratio of the ESR signal of the thin films is better than that of the SWCNT transistor in the absence of dielectric loss due to wirings and electrodes. The solid lines in Fig. 3a show the ESR signal of the SWCNT thin film at 15, 60, and 290 K. At each temperature, the *g* value was found to be 2.0029 ± 0.00002. The Δ*H*_pp_ values were found to be 0.36 ± 0.03 mT at 15 K, 0.33 ± 0.03 mT at 60 K, and 0.50 ± 0.05 mT at 290 K. The open symbols in Fig. 3a show the least-squares fits with a Lorentzian lineshape for the observed ESR signals. The fitting data with a single Lorentzian component explain the experimental results very well at all measured temperatures. In previous ESR studies, a complicated Dysonian lineshape was observed because of the skin effects of microwave absorption due to bulk or thick-film samples^33^,^34^,^38^,^41^,^42^. In contrast to these previous studies, the use of thin films in the present study makes it possible to avoid such skin effects and to perform a precise ESR analysis using a simple Lorentzian lineshape, as described below.

Next, we discuss the detailed temperature dependence of the ESR signal of the SWCNT thin film by presenting the spin susceptibility *χ* and the ESR linewidth. Figure 3b and its inset show the temperature dependence of *χ* and the inverse spin susceptibility 1/*χ*, respectively. The *χ* value was evaluated by integrating the ESR signal twice and comparing it with the Mn^2+^ marker sample. The solid lines in Fig. 3b and its inset show the least-squares fits with the Curie law for the observed *χ* and 1/*χ*, respectively. The fitting curves explain the experimental results very well, which show that *χ* clearly obeys the Curie law. Therefore, we further confirm that the observed ESR signal originates from magnetically isolated spin species, not from Pauli components due to charge carriers, which is consistent with the isolated spin formation at atomic vacancies in the SWCNT thin film^10^,^44^. We evaluated the spin concentration of the SWCNT thin film by calculating *N*_spin_ and considering the volume of the film. The spin concentration was found to be 1.1 × 10^18^ cm^−3^. The average distance between spins was evaluated to be 9.7 nm based on this concentration.

The temperature dependence of the ESR linewidths of the SWCNT thin film is shown in Fig. 3c, where the full width at half maximum of the ESR linewidth *ΔH*_1/2_ is plotted as a function of temperature. In the high temperature region above approximately 70 K, *ΔH*_1/2_ increases with increasing temperature. This behavior can be explained by the decrease in spin-lattice relaxation time *T*_1_ of the vacancies’ spins because the *T*_1_ value can be shortened by phonon modes in the SWCNTs at high temperatures and an ESR linewidth with a Lorentzian lineshape is proportional to 1/*T*_1_, that is, *ΔH*_1/2_ ∝ 1/*T*_1_ (ref. 45). The effects of spin-lattice relaxation on the ESR linewidth is consistent with the observed Lorentzian ESR lineshape at high temperatures. However, in the low temperature region below approximately 70 K, *ΔH*_1/2_ decreases with increasing temperature. This behavior can be explained by the motional narrowing of the ESR linewidth due to the motion of the vacancies’ spins (the motion of vacancies) in the SWCNT thin film because the DFT study showed that bond alternation at vacancies can occur at low energies and cause the motion of vacancies^10^. The motional narrowing of the ESR linewidth is consistent with the observed Lorentzian ESR lineshape at low temperatures.

### Anisotropy of the spin-orbital interaction in SWCNTs

**Figure 4:**
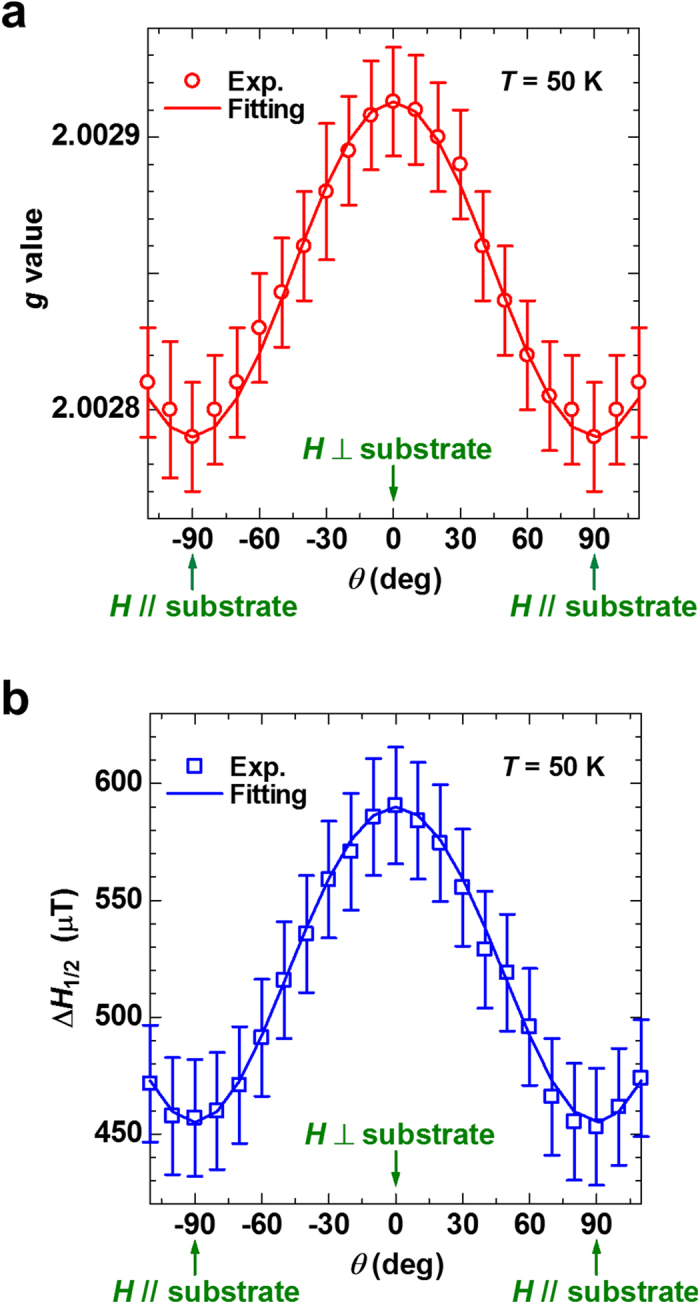
Anisotropy of the ESR signal of the SWCNT thin film. Angular dependence of the *g* value (**a**) and **Δ***H*_1/2_ (**b**) of the ESR signal of the SWCNT thin film on *H* relative to the substrate at 50 K.

Finally, we present the anisotropy of the ESR signal in the direction of the external magnetic field *H* relative to the substrate to investigate the anisotropy of the spin-orbital interaction in the SWCNT thin film. The measurements were performed at 50 K to improve the SN ratio of the ESR signal. Figure 4a,b show the anisotropy of the *g* value and *ΔH*_1/2_ of the ESR signal, respectively, where *θ* is defined as the angle between *H* and the normal of the substrate (Fig. 1a). Clear anisotropy of the *g* value and *ΔH*_1/2_ is observed for the first time in the ESR studies of CNTs. Solid lines in Fig. 4a,b show the least-squares fits with the following fitting curves for the observed *g* value and *ΔH*_1/2_, respectively:


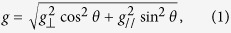



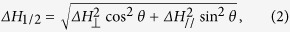


where 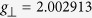 and 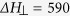 μT at *H* perpendicular to the substrate and 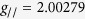 and 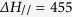 μT at *H* parallel to the substrate. The fitting curves explain the experimental results very well, which show the uniaxial anisotropy of the *g* value and *ΔH*_1/2_ of the SWCNT thin film. Let *λ*, ***L***, and ***S*** be the spin-orbit coupling constant, the orbital angular momentum, and the spin angular momentum of the vacancies’ spins, then the observed anisotropy reveals an anisotropy of the spin-orbit interaction *W*_*LS*_ = *λ*(***L***·***S***) of the vacancies’ spins with respect to the surface of the SWCNTs because the second-order perturbation of *W*_*LS*_ determines the *g* value and *W*_*LS*_ also causes the spin-lattice relaxation affecting the ESR linewidth^46^. We comment that the observed anisotropy is qualitatively consistent with that of graphite^47^, which further confirms that the origin of the observed ESR signal is not due to extrinsic impurities, such as catalysts etc., and/or tube ends of the SWCNTs.

In conclusions, we performed an ESR study of the SWCNT transistors under device operation to investigate the relation between the spin states and the device performance. We demonstrated electrically induced ambipolar spin vanishments in the SWCNTs from a microscopic viewpoint for the first time. This result means the non-magnetization of atomic vacancies in the SWCNT transistor, which is formed by the antiferromagnetic interactions between spins of vacancies and electrically induced electrons in positive *V*_G_ region and by the discharge of the vacancies’ spins in negative *V*_G_ region. The ambipolar spin vanishments would contribute the improvement of transport properties of CNTs because of greatly reduced carrier scatterings due to magnetic interactions. The non-observation of an ESR signal due to electrically induced charges is ascribed to the formation of TLL states. We further present detailed microscopic properties of atomic vacancies, such as spin concentration, spin-lattice relaxation time, motion of vacancies, and anisotropy of spin-orbit interaction in the SWCNT thin film. In particular, an anisotropic ESR signal was firstly observed for the SWCNT thin film. The microscopic information in addition to the ambipolar spin vanishments would be extremely useful for not only understanding material properties of SWCNTs but also fabricating high-performance SWCNT devices.

## Methods

### Fabrication of SWCNT thin films and transistors

We used semiconducting SWCNTs purified from SWCNTs produced by the Arc discharge method (Meijyo Nanocarbon Co., Arc SO). SWCNTs were dispersed into 1 or 2% deoxycholate sodium salt (DOC) solutions, and high-quality semiconducting SWCNTs were obtained through a density gradient ultracentrifugation (DGU) process^25^,^26^,^27^. The obtained SWCNT solutions were carefully washed several times with methanol, hot water, and toluene, and the thin films with 300 nm thickness were fabricated on the substrate. The diameters of the SWCNTs measured by an atomic force microscope were 1.37–1.4 nm. The detailed purification methods are described elsewhere^25^,^26^,^27^.

The SWCNT transistors were fabricated using a nonmagnetic quartz substrate (Iiyama Precision Glass Co., Ltd.). The dimension of the quartz substrate was 30 mm × 3 mm × 1 mm. Thin films of the SWCNTs with the dimension of 15 mm × 2 mm × 300 nm were fabricated on the substrate. The source, drain, and gate electrodes of Ni (3 nm)/Au (47 nm) were fabricated with a vapor-deposition method on the substrate containing the SWCNTs. The source and drain electrodes had a channel length of 0.5 mm and a channel width of approximately 15 mm. We utilized an ion-gel dielectric, a special class of solid polymer electrolyte formed by the ionic liquid 1-ethyl-3-methylimidazolium bis(trifluoromethylsulfonyl)imide ([EMIM][TFSI]) and a gelator ABA-type triblock copolymer poly(styrene-*b*-methylmethacylate-*b*-styrene) (PS-PMMA-PS), which shows large EDL capacitance and high ionic conductivity^20^,^21^. The EDL capacitances are generally very large (~10–100 μF cm^−2^), leading to significant charge accumulation with low voltage and high on/off current ratios^20^,^21^. The details of the ion gels have been described in the literature^17^,^18^,^19^,^20^,^21^,^22^,^23^,^24^. Finally, the ion-gel film was fabricated on the substrate containing the SWCNTs by drop-casting, completing the transistor fabrication. The fabricated devices were sealed into ESR sample tubes under vacuum conditions or under He exchange gas at 100 torr after wirings.

### ESR and transfer characteristic measurements

ESR measurements were performed using a JEOL JES-FA200 X-band spectrometer under vacuum conditions or under He exchange gas at 100 torr. The *g* factor and linewidth of the ESR signals were calibrated using a standard Mn^2+^ marker sample. The *χ* value was evaluated by integrating the ESR signal twice and comparing it with the Mn^2+^ marker sample. The *N*_spin_ value was evaluated by integrating the ESR signal twice and by comparing *N*_spin_ with that of the Mn^2+^ marker sample assuming the Curie law. The absolute value of *N*_spin_ of the Mn^2+^ marker sample was calculated using a solution (220 μL) of 4-hydroxy-2,2,6,6-tetramethylpiperidin-1-oxyl (TEMPOL) as a standard. The calibration of the *g* factor was performed using a software program from the JEOL ESR system considering high second-order correction to the effective resonance field. Its correctness was also confirmed using 2,2-diphenyl-1-picrylhydrazyl (DPPH) as another standard sample. Simultaneous measurements of ESR and the device characteristics were performed using a Keithley 2612A source meter.

## Additional Information

**How to cite this article**: Matsumoto, D. *et al.* Electrically induced ambipolar spin vanishments in carbon nanotubes. *Sci. Rep.* **5**, 11859; doi: 10.1038/srep11859 (2015).

## References

[b1] Javey A., Guo J., Wang Q., Lundstrom M. & Dai H. Ballistic carbon nanotube field-effect transistors. Nature 424, 654–657 (2003). 12904787 10.1038/nature01797

[b2] Dresselhaus M. S. New tricks with nanotubes. Nature 391, 19–20 (1998).

[b3] Ouyang M., Huang J. L. & Lieber C. M. Fundamental Electronic Properties and Applications of Single-Walled Carbon Nanotubes. Acc. Chem. Res. 35, 1018–1025 (2002). 12484789 10.1021/ar0101685

[b4] McEuen P. L. Carbon-based electronics. Nature 393, 15–16 (1998).

[b5] Avouris P. Molecular Electronics with Carbon Nanotubes. Acc. Chem. Res. 35, 1026–1034 (2002). 12484790 10.1021/ar010152e

[b6] Graham A. P. *et al.* How do carbon nanotubes fit into the semiconductor roadmap? Appl. Phys. A: Mater. Sci. Process. 80, 1141–1151 (2005).

[b7] Franklin A. D. & Chen Z. Length scaling of carbon nanotube transistors. Nature Nanotech. 5, 858–862 (2010). 10.1038/nnano.2010.220 21102468

[b8] Avouris P. & Martel R. Progress in Carbon Nanotube Electronics and Photonics. MRS Bull. 35, 306–313 (2010).

[b9] Vijayaraghavan A. *et al.* Toward Single-Chirality Carbon Nanotube Device Arrays. ACS Nano 4, 2748–2754 (2010). 20408580 10.1021/nn100337t

[b10] Ma Y., Lehtinen P. O., Foster A. S. & Nieminen R. M. Magnetic properties of vacancies in graphene and single-walled carbon nanotubes. New J. Phys. 6, 68 (2004).

[b11] Marumoto K., Kuroda S., Takenobu T. & Iwasa Y. Spatial Extent of Wave Functions of Gate-Induced Hole Carriers in Pentacene Field-Effect Devices as Investigated by Electron Spin Resonance. Phys. Rev. Lett. 97, 256603 (2006). 17280376 10.1103/PhysRevLett.97.256603

[b12] Marumoto K. *et al.* Microscopic mechanisms behind the high mobility in rubrene single-crystal transistors as revealed by field-induced electron spin resonance. Phys. Rev. B. 83, 075302 (2011).

[b13] Tsuji M. *et al.* Two-dimensional magnetic interactions and magnetism of high-density charges in a polymer transistor. Appl. Phys. Lett. 102, 133301 (2013).

[b14] Takahashi Y. *et al.* Electron Spin Resonance Study of Organic Interfaces in Ion Gel-Gated Rubrene Single-Crystal Transistors. Appl. Phys. Express 6, 041603 (2013).

[b15] Marumoto K., Fujimori T., Ito M. & Mori T. Charge Formation in Pentacene Layers During Solar-Cell Fabrication: Direct Observation by Electron Spin Resonance. Adv. Energy Mater. 2, 591–597 (2012).

[b16] Nagamori T. & Marumoto K. Direct Observation of Hole Accumulation in Polymer Solar Cells during Device Operation using Light-Induced Electron Spin Resonance. Adv. Mater. 25, 2362–2367 (2013). 23447406 10.1002/adma.201204015

[b17] Siddons G. P., Merchin D., Back J. H., Jeong J. K. & Shim M. Highly Efficient Gating and Doping of Carbon Nanotubes with Polymer Electrolytes. Nano Lett. 4, 927–931 (2004).

[b18] Shimotani H. *et al.* Gate capacitance in electrochemical transistor of single-walled carbon nanotube. Appl. Phys. Lett. 88, 073104 (2006).

[b19] Panzer M. J. & Frisbie C. D. Exploiting Ionic Coupling in Electronic Devices: Electrolyte-Gated Organic Field-Effect Transistors. Adv. Mater. 20, 3177–3180 (2008).

[b20] Cho J. H. *et al.* Printable ion-gel gate dielectrics for low-voltage polymer thin-film transistors on plastic. Nature Mater. 7, 900–906 (2008). 18931674 10.1038/nmat2291

[b21] Lee J. *et al.* Ion Gel-Gated Polymer Thin-Film Transistors: Operating Mechanism and Characterization of Gate Dielectric Capacitance, Switching Speed, and Stability. J. Phys. Chem. C 113, 8972–8981 (2009).

[b22] Zaumseil J., Ho X., Guest J. R., Wiederrecht G. P. & Rogers J. A. Electroluminescence from Electrolyte-Gated Carbon Nanotube Field-Effect Transistors. ACS Nano 3, 2225–2234 (2009). 19634895 10.1021/nn9005736

[b23] Yomogida Y. *et al.* Ambipolar Organic Single-Crystal Transistors Based on Ion Gels. Adv. Mater. 24, 4392–4397 (2012). 22729886 10.1002/adma.201200655

[b24] Ha M. *et al.* Aerosol Jet Printed, Low Voltage, Electrolyte Gated Carbon Nanotube Ring Oscillators with Sub-5 *μ*s Stage Delays. Nano Lett. 13, 954–960 (2013). 23394463 10.1021/nl3038773

[b25] Yanagi K., Miyata Y. & Kataura H. Optical and Conductive Characteristics of Metallic Single-Wall Carbon Nanotubes with Three Basic Colors; Cyan, Magenta, and Yellow. Appl. Phys. Express 1, 034003 (2008).

[b26] Sato Y. *et al.* Chiral-Angle Distribution for Separated Single-Walled Carbon Nanotubes. Nano Lett. 8, 3151–3154 (2008). 18729412 10.1021/nl801364g

[b27] Yanagi K. *et al.* Transport Mechanisms in Metallic and Semiconducting Single-Wall Carbon Nanotube Networks. ACS Nano 4, 4027–4032 (2010). 20593841 10.1021/nn101177n

[b28] Tsuji M. *et al.* Electron Spin Resonance Study of Interface Trap States and Charge Carrier Concentration in Rubrene Single-Crystal Field-Effect Transistors. Appl. Phys. Express 4, 085702 (2011).

[b29] Tanaka H. *et al.* Electron spin resonance observation of charge carrier concentration in organic field-effect transistors during device operation. Phys. Rev. B 87, 045309 (2013).

[b30] Havlicek M. *et al.* Magnetic phase transition for defect induced electron spins from fully metal–semiconductor separated SWCNTs. Phys. Status Solidi B 249, 2562–2567 (2012).

[b31] Dóra B. *et al.* Electron Spin Resonance Signal of Luttinger Liquids and Single-Wall Carbon Nanotubes. Phys. Rev. Lett. 101, 106408 (2008). 18851238 10.1103/PhysRevLett.101.106408

[b32] Kosaka M., Ebbesen T. W., Hiura H. & Tanigaki K. Annealing Effect on Carbon Nanotubes. An ESR study. Chem. Phys. Lett. 233, 47–51 (1995).

[b33] Petit P., Jouguelet E., Fischer J. E., Rinzler A. G. & Smalley R. E. Electron spin resonance and microwave resistivity of single-wall carbon nanotubes. Phys. Rev. B 56, 9275–9278 (1997).

[b34] Claye A. S., Nemes N. M., Jánossy A. & Fischer J. E. Structure and electronic properties of potassium-doped single-wall carbon nanotubes. Phys. Rev. B 62, R4845–R4848 (2000).

[b35] Salvetat J.-P., Fehér T., L’Huillier C., Beuneu F. & Forró L. Anomalous electron spin resonance behavior of single-wall carbon nanotubes. Phys. Rev. B 72, 075440 (2005).

[b36] Náfrádi B. *et al.* Electron spin resonance of single-walled carbon nanotubes and related structures. Phys. Status Solidi B 243, 3106–3110 (2006).

[b37] Musso S. *et al.* Low temperature electron spin resonance investigation on SWNTs after hydrogen treatment. Diamond Relat. Mater. 15, 1085–1089 (2006).

[b38] Likodimos V., Glenis S., Guskos N. & Lin C. L. Antiferromagnetic behavior in single-wall carbon nanotubes. Phys. Rev. B 76, 075420 (2007).

[b39] Corzilius B. *et al.* SWNT probed by multi-frequency EPR and nonresonant microwave absorption. Phys. Status Solidi B 245, 2251–2254 (2008).

[b40] Kombarakkaran J. & Pietraβ, T. Electron spin resonance studies of hydrogen adsorption on single-walled carbon nanotubes. Chem. Phys. Lett. 452, 152–155 (2008).

[b41] Ferrer-Anglada N., Monge A. A. & Roth S. Electron spin resonance on single-walled carbon nanotubes obtained from different sources. Phys. Status Solidi B 247, 2823–2826 (2010).

[b42] Havlicek M. *et al.* Electron spin resonance from semiconductor–metal separated SWCNTs. Phys. Status Solidi B 247, 2851–2854 (2010).

[b43] Szirmai P. *et al.* Density of states deduced from ESR measurements on low-dimensional nanostructures; benchmarks to identify the ESR signals of graphene and SWCNTs. Phys. Status Solidi B 248, 2688–2691 (2011).

[b44] Rice W. D. *et al.* Enhancement of the Electron Spin Resonance of Single-Walled Carbon Nanotubes by Oxygen Removal. ACS Nano 6, 2165–2173 (2012). 22324937 10.1021/nn204094s

[b45] Havlicek M. *et al.* Indirect exchange interaction in fully metal-semiconductor separated single-walled carbon nanotubes revealed by electron spin resonance. Phys. Rev. B 86, 045402 (2012).

[b46] Pake G. E. Paramagnetic Resonance (W. A. Benjamin, Inc., New York, 1962) pp. 126 128.

[b47] Wagoner G. Spin Resonance of Charge Carriers in Graphite. Phys. Rev. 118, 647–653 (1960).

